# ChatGPT: these are not hallucinations – they’re fabrications and falsifications

**DOI:** 10.1038/s41537-023-00379-4

**Published:** 2023-08-19

**Authors:** Robin Emsley

**Affiliations:** Editor, Schizophrenia, https://www.nature.com/npjschz/

The artificial intelligence (AI) system, Chat Generative Pre-trained Transformer (ChatGPT), is considered a promising, even revolutionary tool and its widespread use in health care education, research, and practice is predicted to be inevitable^1^. Like so many others I was keen to test the capabilities of ChatGPT as an aid to scientific writing. An opportunity arose with a study I was planning on an existing dataset (structural MRI brain changes associated with antipsychotic treatment). After registering with OpenAI online, I provided basic information on the dataset and planned study and requested suggestions for the study methodology. These were promptly provided as broad stroke proposals about identifying a research question, hypotheses, suitable outcomes etc. The interaction felt eerily interpersonal and the chatbot’s demeanour was cordial and helpful, even eager. My questions relating to overall methodology produced mostly sensible suggestions, although largely predictable and somewhat mundane. I had difficulty in getting beyond the general responses that were provided for the statistical analysis plan. Specific questions were deflected with generalisations and recommendations to consult a statistician. At that stage I was unimpressed, but there were no concerns.

However, after that the problems emerged. I asked which brain regions would be of particular interest in relation to antipsychotic treatment. The thalamus was an unexpected suggestion, so I requested supportive literature. Five references were duly supplied, including publications by established researchers in reputable journals. (These references are provided as Supplement 1). By this time my level of enthusiasm had risen considerably as new possibilities for the analysis emerged based on these heretofore undiscovered studies. My next step was to source these publications via PubMed. The first reference was to a well-known longitudinal study reporting brain changes over time - but it was inappropriate, as it was not focussed on the thalamus. I was unable to trace three of the four remaining references, whether I searched by author names, manuscript title or journal details. Similarly, with Google Scholar I was not able to identify the articles. Entering the Digital Object Identifier (DOI) number in my searches took me to totally unrelated publications. Becoming increasingly uneasy, I questioned ChatGPT about previous studies whose content I was familiar with, including my own, as a test of accuracy. Some of the answers provided were patently incorrect. The problem therefore goes beyond just creating false references. It includes falsely reporting the content of genuine publications. Thus, while most attention to date has focussed on the production of false references as these are the easiest to detect, the veracity of any content inputs provided by ChatGPT cannot be trusted. The cause of these falsifications has been linked to a disturbance in language production, with probabilistic outputs based on estimates of semantic similarity. This allows informed guesses, with bits of false information being mixed with factual information^2^.

Alarmed, I assumed I had done something wrong. I instructed ChatGPT to check one of the incorrect references. I received an apology for the mistake and was provided with the “correct” reference. However, this one was also incorrect. And so too with the third attempt. On chatting to colleagues and checking the online literature it became apparent that my experience wasn’t unique and that the problem is widespread^3^. One study investigating the frequency of so-called AI hallucinations in research proposals generated by ChatGPT found that out of 178 references cited, 69 did not have a DOI, 28 of which were found not to exist^4^. Another study investigating the authenticity and accuracy of references in medical articles generated by ChatGPT found that of 115 references that were generated, 47% were fabricated, 46% were authentic but inaccurate, and only 7% were authentic and accurate^5^. A further study assessing whether ChatGPT can reliably produce accurate references to supplement manual literature searches reported that of 35 generated citations, only two were real, 12 were similar to actual manuscripts and the remaining 21 were seemingly plausible but in fact a mix of multiple actual manuscripts^2^. This is outrageous. While concerns and cautions have been expressed in the rapidly emerging literature, I would have expected a stronger response. How could the use of such a profoundly defective tool as this be permitted without public outcry and calls for prohibition of its further use in the research environment? The phenomenon has been charitably referred to as hallucinations. This is a misnomer. Hallucinations are false perceptions. What I experienced were fabrications and falsifications. The Office of Research Integrity of the United States Department of Health and Human Services defines fabrication as the making up data or results and falsification as the manipulation, changing or omitting of data or results such that the research is not accurately represented (https://ori.hhs.gov/definition-research-misconduct). Or, if the absence of malicious intent is assumed, confabulations would be a better description, as has been proposed^2^. In any event, the potential consequences are dire. The risk is compounded firstly by the believability of the falsifications, even deceiving established scientists^6^, and secondly by its tendency to “double down” convincingly when confronted with these inaccuracies^5^.

My sobering experience spurred me to write this editorial, to alert others to the dangers. And of course, who better to assist than ChatGPT? My request was: “Write an editorial of 2000 words with references for a scientific journal on schizophrenia about using ChatGPT in research. It should be interesting and controversial, highlighting the pros and cons of its use.” The first response ignored my request for references, so I asked: “Include up to 6 references.” A list of 6 references was provided but not cited in the text. I asked: “Cite these references in the text” The references were again provided but not the citations in the text. I asked: “re-write the editorial citing the references in the appropriate places in the text”. This was provided, but this time the reference list was incomplete. There seemed to be a decided reluctance to perform the task, perhaps because of our previous interaction regarding false references. I asked: “Why did you not complete the reference list?” the response: “I apologize for the incomplete reference list in the previous response. Here is the complete reference list for the editorial.” And again, “I apologize for any inconvenience caused by the initial incomplete reference list.” The ChatGPT generated editorial, together with citations and references are provided as Supplement 2. Predictably, fact checking indicated problems. The first reference was inappropriate and ref. ^5^ was a flagrant example of a fictitious reference.

So, use ChatGPT at your own peril. Just as I would not recommend collaborating with a colleague with pseudologia fantastica, I do not recommend ChatGPT as an aid to scientific writing. While the global move to regulating AI seems to be largely driven by its perceived extinction risk to humanity, it seems to me that a more immediate threat is the infiltration into the scientific literature of masses of fictitious material.

Watch Prof Emsley discussing this editorial: https://vimeo.com/844688249/56fcfd10d6.

### Supplementary information

Supplement 1

Supplement 2
