# Supplementary material for: ChatGPT: these are not hallucinations – they’re fabrications and falsifications
Source: Schizophrenia (Heidelb). 2023 Aug 19;9(1):52. doi: 10.1038/s41537-023-00379-4 (PMC10439949; doi:10.1038/s41537-023-00379-4)
Supplement: Supplementary file 1 — Supplement 1 [file 41537_2023_379_MOESM1_ESM.docx]

**Supplement 1:**

***My question to ChatGPT:***

Provide references for thalamus changes and antipsychotic treatment.

***ChatGPT response:***

Here are a few references on thalamus changes and antipsychotic treatment:

1. Ho BC, Andreasen NC, Nopoulos P, Arndt S, Magnotta V, Flaum M. Progressive structural brain abnormalities and their relationship to clinical outcome: a longitudinal magnetic resonance imaging study early in schizophrenia. Arch Gen Psychiatry. 2003;60(6):585-594. doi:10.1001/archpsyc.60.6.585
2. Molina V, Reig S, Sanz J, Palomo T, Benito C, Sánchez J, Pascau J, Desco M. Increase in gray matter and cerebrospinal fluid volume after remission in schizophrenia. Schizophr Res. 2005;77(2-3):181-188. doi:10.1016/j.schres.2005.03.009
3. Moncrieff J, Leo J. A systematic review of the effects of antipsychotic drugs on brain volume. Psychol Med. 2010;40(9):1409-1422. doi:10.1017/S0033291709992297
4. Mitelman SA, Brickman AM, Shihabuddin L, et al. A comprehensive assessment of gray and white matter volumes and their relationship to outcome and severity in schizophrenia. Neuroimage. 2007;37(2):449-462. doi:10.1016/j.neuroimage.2007.05.019
5. Baiano M, David A, Versace A, Churchill R. Antipsychotic medication and brain morphology in schizophrenia: a systematic review of the literature. Psychol Med. 2007;37(9):1175-1184. doi:10.1017/S0033291707000138

These studies suggest that thalamus volume changes may occur in patients with schizophrenia treated with antipsychotic medication, although the exact nature of these changes and their relationship to clinical outcomes may vary. It's important to note that further research is needed to fully understand the effects of antipsychotic treatment on the thalamus and other brain regions in schizophrenia.
